# Impact of Layer Thickness and Storage Time on the Properties of 3D-Printed Dental Dies

**DOI:** 10.3390/ma14030509

**Published:** 2021-01-21

**Authors:** Aya Sabbah, Georgios Romanos, Rafael Delgado-Ruiz

**Affiliations:** 1 Department of Oral Biology and Pathology, School of Dental Medicine, Stony Brook University, Stony Brook, NY 11794, USA; ayasabbah@gmail.com; 2 Department of Periodontology, School of Dental Medicine, Stony Brook University, Stony Brook, NY 11794, USA; Georgios.Romanos@stonybrookmedicine.edu; 3 Department of Prosthodontics and Digital Technology, School of Dental Medicine, Stony Brook University, Stony Brook, NY 11794, USA

**Keywords:** dentistry, 3D-printing, printed dies, dimensional stability, surface roughness, stereolithography

## Abstract

The purpose of this study was to evaluate the effect of printing layer thickness on the repeatability and surface roughness of 3D-printed dies and detect the effect of layer thickness and storage time on the dimensional stability of 3D-printed dies. One stereolithography (STL) file of an upper molar prepared for a full ceramic crown was used to print three groups of dies: 25 µm, 50 µm, and 100 µm. Repeatability was evaluated by linear and area measurements with a digital caliper and a digital metrology microscope. Dimensional stability was analyzed at 3 weeks, 6 months, and 1 year of storage time. Surface roughness parameters were measured with a 3D confocal laser scanning microscope. Statistics were completed using one-way analysis of variance and Tukey’s post hoc tests, *p* < 0.05. Printing time decreased as layer thickness increased. All groups showed high repeatability and comparable surface roughness while showing differences in their linear dimensions and surface areas. At the 3 week storage interval, dimensional changes were observed in all groups. Within this experimental study’s constraints, it can be concluded that changing the 3D-printing layer thickness does not affect the repeatability or the surface roughness of the product; meanwhile, changes to the layer thickness and storage time influence the dimensional stability of 3D-printed dies.

## 1. Introduction

A well-fitted dental prosthesis relies on reducing errors during each step of its fabrication [1]. Traditionally, the fabrication process goes through a sequence of manual steps, starting with the impression and ending with the insertion of an accurate, functional, and esthetic restoration. Errors can be introduced at any point in the conventional workflow [2]. The digitalization of the conventional processes using digital dentistry techniques can contribute to reducing errors, steps, and effort, as well as the reduction of wasted materials [3]. 

In digital dentistry, additive (3D-printing) and subtractive manufacturing techniques are used to fabricate a wide range of objects [4]. The American Society for Testing and Materials (ASTM) has defined additive manufacturing (AM) as a method of uniting materials to produce objects originated from 3D model data, layer superimposed over layer. [5]. The ASTM international committee on AM technologies has determined seven AM categories: stereolithography (SLA), material jetting (MJP), material extrusion or fused deposition modelling (FDM), binder jetting, powder bed fusion (PBF), sheet lamination, and direct energy deposition [5]. 

Products of AM in dentistry include surgical guides, orthodontic aligners, temporary restorations, denture bases and teeth, dental models, dental implants, and dental dies [6,7]. The dental die is a positive reproduction of a tooth inside a working or studying model [8]. Maintaining the integrity and the volumetric stability of the die throughout its storage time is crucial in confirming the adaptation of any final restoration over the tooth that the dental die represents [8]. Furthermore, having a dimensionally stable die allows for the achievement of precise occlusal and interproximal contacts [9]. 

At present, a completely digital workflow is possible in clinical dentistry, from digital impression through manufacturing production to final framework. Additionally, a digital workflow shows good clinical reliability and excellent patient feedback [10,11]. 

If the die dimensions change, the marginal gap can increase, the cement space can be altered, and the final crown could not fit adequately. This may result, clinically, in food trapping, caries, crown loosening, and crown failure [12]. Traditionally, dies are fabricated in type IV/V die stone, epoxy resins, or metals. From these material options, type IV die stone has been the gold standard due to its detail reproducibility and dimensional stability [13]. However, its strength can be compromised if the water–powder ratio is altered [14,15].

Dental dies can also be fabricated by 3D-printing. The 3D-printing of dental dies involves scanning a prepared tooth with an intraoral scanner (the direct technique) or by scanning an impression or a cast with a laboratory scanner (the indirect technique) [16,17,18]. The data are converted into a standard tessellation language (STL) file that is edited using the appropriate software to create a digital model sent for manufacturing by additive (3D-printing) or subtractive (milling) methods. The additive technique builds up an object layer-by-layer and allows for the fabrication of more complex structures than subtractive methods [19,20].

Among the several additive manufacturing techniques is stereolithography (SLA), a well-known technique in the AM industry that has been used in medical devices like hearing aids It is a widely preferred method in dentistry due to its precision and small part accuracy [21,22]. The SLA technique uses a unique curing source, a compatible resin, and a typical polymerization reaction. Additionally, SLA offers the option of printing with different layer thicknesses, which may impact the final surface characteristics [23,24]. 

The printed dies should keep stable dimensions and maintain a reliable reference to allow for laboratory work, the clinician’s evaluation, and ample storage time [25]. There are no studies elaborating on the effect of changing layer thickness on the repeatability, surface roughness, and dimensional stability of 3D-printed structures (dies) after different amounts of storage time.

The purpose of this study was to evaluate the effect of printing layer thickness on repeatability and surface roughness and to detect the effect of layer thickness and storage time on the dimensional stability of 3D-printed dies.

## 2. Materials and Methods 

**Figure 1:**
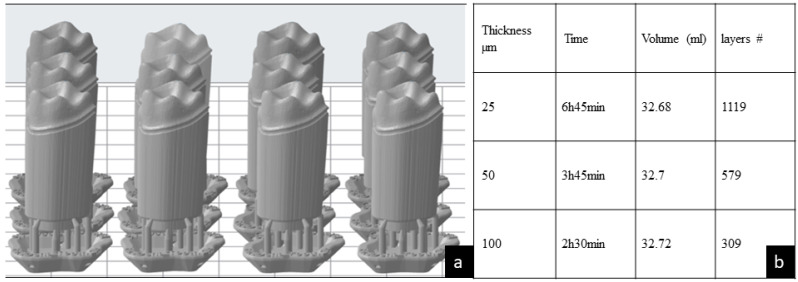
(**a**) Lateral view of the standard tessellation language (STL) file of 12 dental dies presented in the Preform software program. (**b**) The printing time of the 12 dental dies decreases as the printed layer thickness increases. The volume of the print remains relatively the same. The number of layers decreased gradually by increasing the thickness from group 25, to 50, to 100 µm.

A typodont (ModuPRO Pros, Acadental Inc., Lenexa, KS, USA) was selected to prepare a first upper left molar. The preparation characteristic for a full ceramic crown was completed as follows: 360-degree shoulder, 1 mm margin width, 6°-degrees tapered walls, an occlusal reduction of 2 mm, and round line angles [26]. The preparation was scanned in a Dental Wings^®^7 Series laboratory scanner (Dental Wings^®^, Montreal, QC, Canada) following the protocol described by Alexandru et al. [7]. The file was saved as an STL file, the margin limits were confirmed and marked, the emergence profile was maintained, and an 18 mm die stand was digitally created to allow for printing of the die without affecting the margin characteristics. The file was exported to Preform^®^ software Version 3.0.1. (Formlabs, Inc., Somerville, MA, USA), which controls the printing settings (e.g., orientation, layer thickness, and printing material) in the Form2 3D-printer (Formlabs, Inc., Somerville, MA, USA). The settings included supports of 0.6 mm point size, 0.85 density, a 3.5 mm height above the supporting raft, and 1 mm raft thickness. Using this Preform^®^ software, three test groups were oriented at a 90-degree angle, differing only in layer thickness as follows: Group 25 µm (n = 12), Group 50 µm (n = 12), and Group 100 µm (n = 12), as seen in Figure 1a. 

Each group was printed independently in the Form2 printer. The printing was set by photopolymerization at 405 nm UV light (Formlabs Inc., Somerville, MA, USA) using the V3 (FLGPR03) grey resin (Formlabs Inc., Somerville, MA, USA). 

After printing, the dies were immersed and washed by agitation in the Form Wash^®^ (Formlabs Inc., Somerville, MA, USA), using 75% Isopropyl Alcohol (IPA) for 30 min. Afterward, the attached supports were removed and the dies were air-dried at room temperature. Finally, the dies were placed in a Form Cure^®^ (Formlabs Inc., Somerville, MA, USA) postcuring UV chamber for 60 min at 60 °C and 405 nm UV light [27]. 

The total printing time, volume of resin required, and number of layers for each group of 12 dies were obtained with the Preform^®^ Software, as seen in Figure 1b.

To evaluate the repeatability, the linear and surface area dimensions were evaluated.

**Figure 2:**
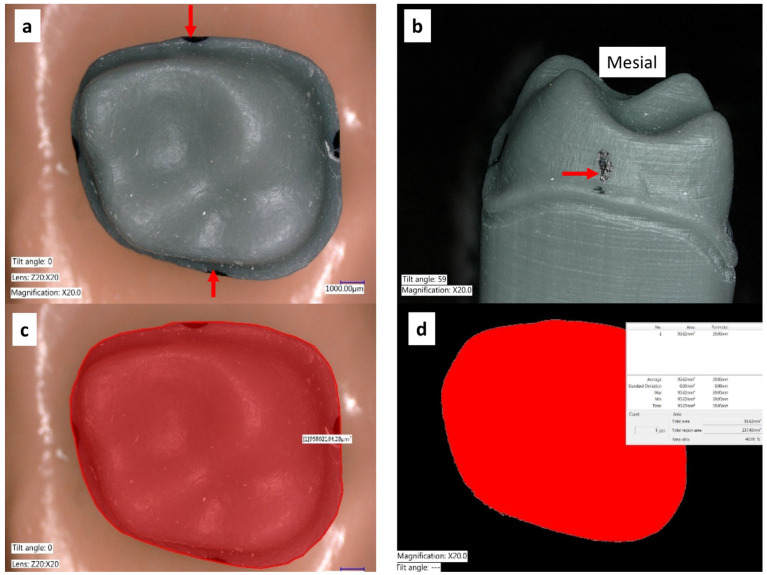
(**a**) Most prominent points of the die contours were marked. (**b**) A direct view of the mesial view showing the most prominent point. (**c**) The outline of the margin contour observed from an occlusal (top) view was traced (**d**) The area inside the margin was obtained and the surface area was calculated automatically by using the color difference detection function of the VHX-6000 microscope software.

**Linear dimensions:** Each die was marked with a permanent marker (Sharpie^®^, Shelbyville, TN, USA) with a 1 mm diameter tip at the maximum contour of the margin at all the surfaces. The mesial–distal (M–D) distances between the mesial and distal points expressed in millimeters (mm) were measured with a digital caliper (Keyence VHX-6000 digital microscope, KEYENCE, Itasca, IL, USA) by the same calibrated operator and recorded for each die in the three groups. The procedure was completed as follows: a digital mark was created from one point to the other for each die, the point recognition software of the microscope was used to detect the marks, and the measurement from each die was recorded (Figure 2a).

**Surface area dimensions:** The surface area was measured using the same digital metrology microscope (Keyence VHX-6000, KEYENCE, Itasca, IL, USA) at a magnification of 50x. An axial view of the occlusal surface was recorded. A digital algorithm was used to outline the margin, and the surface area (expressed in mm^2^) contained within the margin outline was calculated (Figure 2b).

**Dimensional stability:** The dimensional stability of the dies over time was evaluated by repeated measurements of the linear and the surface area dimensions immediately after printing (T0), after three weeks (T1), six months (T2), and at one year (T3). The storage conditions were: a room temperature between 24–27 °C, a relative humidity of 50%, and the samples were maintained in enclosed dark containers. 

**Surface roughness:** Two height parameters (*S_a_, S_z_),* one spatial parameter (S_tr_), one feature parameter (*S_pc_*), and one hybrid parameter *(S_dr_*) were evaluated using a laser digital microscope (Keyence 3D Laser Confocal digital VK-X100, KEYENCE, IL, USA) at magnification 50X. The definitions of the selected surface parameters are as follows:

*S_a_ (µm) Arithmetical Mean Height:* This represents the arithmetic mean of the peaks and valleys of the surface evaluated in different regions of interest.

*S_z_ (µm) Maximum Height*: The maximum peak height and maximum pit depth values within the designated areas of interest.

*S_tr_ Texture Aspect Ratio:* This parameter quantifies texture strength. Values near 1 demonstrate isotropy (directionality) of the surface. Meanwhile, values near 0 demonstrate anisotropy.

*S_pc_ (1/mm) Arithmetic Mean Peak Curvature:* This parameter represents the arithmetic mean of the main curvature of the peaks on the surface.

*S_dr_ Developed Interfacial Area Ratio:* The surface area contributed by the texture as compared to the planar definition area. The *S_dr_* of a completely flat surface is 0.

**Figure 3:**
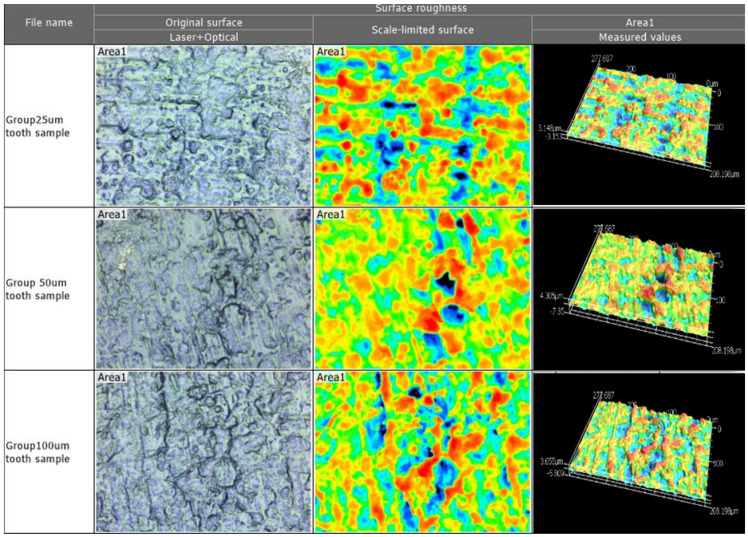
The surface roughness of the tooth die surface for each of the three groups in magnified 3D. Taken by Profile-analyzing Laser Microscope VK-X100 series, Keyence@.

Four areas of 100 µm × 100 µm were randomly located on the proximal surfaces of the dies (buccal, lingual, mesial, or distal) at a 50× magnification (n = 4 × 12 × 3 groups = 144 measures), as seen in Figure 3. Next, using the MultiFile Analyzer^®^ software Version 2.5.0. (KEYENCE, Itasca, IL, USA), the following filtering processes were applied: Missing and weak data were removed using a signal filter, and upper and lower height thresholds were determined by manually selecting the highest and the lowest intensity peaks. To eliminate the effect of the surface curvature, the automatic selection of a flat reference plane was selected. The same filters were applied to all samples using the batch analysis criteria. Mean and standard deviation values were calculated for all parameters for all dies. 

**Statistical analysis:** Sample size was determined to be 12 for each group according to Java Applet Software of Indiana University [28], considering a power of 85% and an alpha error of 5%. Each group’s repeatability was evaluated by using dispersion measures around the means, including standard deviation (STDEV), variance, and interquartile range (IQR). 

Dimensional changes and surface roughness were evaluated using the GraphPad Prism 8.2 software (GraphPad Software^®^ San Diego, CA, USA). The Kolmogorov–Smirnov test was used to confirm the normality of the data and a one-way ANOVA single factor test (α = 0.05) was used to evaluate the differences between the means, followed by multiple Tukey test post hoc comparisons. Statistical significance was considered to be met when the *p*-value was <0.05.

## 3. Results

**Intragroup comparisons (repeatability):** At time zero (T0), each group’s linear dimensions showed minimal within-group variances (0.01 mm^2^) and standard deviations (0.1 mm) (12 dies per group). In terms of surface area, the variances in the measurements were 0.26–0.46 mm^4^, while their standard deviations were 0.5–0.7 mm^2^, indicating sufficient repeatability (Table 1).

**Intergroup comparisons:** At printing time 0, there was a statistically significant difference noted in the linear dimensions (0.15 mm) and surface area (0.77 mm^2^), specifically between the 25 µm and 100 µm groups. The dimensional means were distinctively smaller in the 100 µm group when compared to the 25 µm and 50 µm groups right after printing (*p* < 0.05), as seen in Table 2.

**Dimensional stability over time:** The mean linear dimension and surface area showed expansion over the period of 1 year for all three groups, mainly in the first 6 months, and more precisely in the first 3 weeks, as seen in the surface areas (significantly in the 50 µm and 100 µm groups). See Table 3.

**Surface roughness:** The roughness parameters *S_a_*, *S_z_*, *S_tr_*, *S_pc_*, and *S_dr_* were similar among the groups (*p* = 0.11–0.98), as seen in Table 4.

## 4. Discussion

The purpose of this study was to evaluate the effect of printing layer thickness on the repeatability and surface roughness of 3D-printed dies and the effect of layer thickness and storage time on the dimensional stability of 3D-printed dies. In the present work, the printed dies showed minor differences in their initial dimensions independent of layer thickness. This finding represents excellent repeatability (minimal intragroup variability) [29]. These findings can be explained by the standardization of the printing parameters, including sample orientation, support number, support locations, the resin used for printing, and postprocessing methods, which were the same for all groups. Arnold et al. [30] found that 3D-printing produced a roughness between 0.87 and 4.44 µm, which is higher than casted or milled structures. The differences between the results of Arnold et al. [30] and the results of the present work can be explained by the sample orientation during printing used in the present study (being the same for all samples) and the standardization of microscopy analysis at a high magnification that evaluated the surface of the most external layer but not the layer’s interfaces. Meanwhile, the Arnold et al. study used different printing angles and a single roughness parameter. 

The findings of the present work are in agreement with the study by Shim et al. [31]; in their study, the samples were printed at different angles, and the authors observed that a 90-degree angle provided the lowest dimensional errors (length and thickness), the best surface (lower roughness), and better mechanical properties compared to 0-degree and 45-degree printing angles. 

The dimensional changes suffered by resin 3D-printed structures might be explained by free monomers and oligomers, the spacing between printing layers, and microstructural defects induced during printing (with UV light/laser light printing) [6,32,33]. On the other hand, post-printing dimensional changes can also affect a 3D-printed object in many ways, including dissolution, disintegration, delamination, and swelling, not to mention that all of these are time-dependent [34]. In the case of photopolymers, although they are resistant to solvents, they can suffer swelling, characterized by an increase in volume [34,35].

Gojzewski et al. found that a variable interfacial space of around 11 μm thickness can persist between each 3D-printed layer, resulting in a nonhomogeneous photopolymerization produced by oxygen molecules present in the air and their diffusion over time through the interphases of the printed material [36]. Their results agree with those of the present study, which show that the samples suffered expansion when the follow-up period was increased. Accuracy is essential when working with a dental die where a crown will be designed, fabricated, and evaluated. Any change in the measured area or dimensions should not exceed a range between 40 and 120 µm of discrepancy depending on the restoration’s material [37]. Jang et al. evaluated the fit (marginal and internal) of single crowns fabricated on 3D-printed models. Their results showed that the crowns manufactured on 3D-printed models possessed an inferior fit compared to the crowns fabricated on conventional casts. This can be explained by the potential dimensional changes suffered by 3D-printed structures over time, as presented in the results of the present work [38].

This experimental study has some drawbacks. First, a single resin was used for printing. Therefore, these results could not be generalized to other resins; however, the composition of the grey resin used in this study is comparable to other resin types produced by the same manufacturer. Thus, the results might be extrapolated to more resins from the same manufacturer [39]. Alongside this, a single printer was used, and the printer’s performance may have influenced printing quality. However, the differences between printers, their calibrations, and other limitations in layer thickness (Z-resolution) capabilities could introduce more variability in the results. The potential changes in the mechanical properties of 3D-printed resins over long periods of storage are not well understood. Finally, the influences of room temperature and humidity are variable factors in laboratory and clinical settings; therefore, the results of the present work must be considered applicable to similar storage conditions. The strengths of the present work are the standardization of the printing parameters, the use of a single calibrated printer, the use of a material which is similar to other materials from the same manufacturer, the use of hybrid parameters for the evaluation of the surface roughness, and the use of homogeneous and well-reported storage conditions that allow for the reproduction of the experimental conditions. Therefore, based on the results of the present study, a dental die should be printed with layers of 25 µm thickness, and any precision work needing to be completed or verified on a resin 3D-printed die should be accomplished during the T0 (immediately after printing and processing) and the T1 (three weeks after printing and processing) periods, where the lowest dimensional changes were observed in the 3D-printed dies.

## 5. Conclusions

Within this experimental study’s constraints, it can be concluded that changing the 3D-printing layer thickness does not affect the repeatability or the surface roughness of the product. Meanwhile, changing the layer thickness as well as the storage time influences the dimensional stability of 3D-printed dies.

## Figures and Tables

**Table 1:** The intragroup repeatability of the linear dimensions and surface area. Standard deviation: STDEV; interquartile range: IQR; mesial–distal width: M–D; time 0: T0; variance: V.

Layer Thickness	Linear Dimension			Surface Area		
	Mean ± STDEV (mm)	V (mm^2^)	IQR (mm)	Mean ± STDEV (mm^2^)	V (mm^4^)	IQR (mm^2^)
25 µm	9.63 ± 0.12	0.02	0.15	95.76 ± 0.51	0.28	0.78
50 µm	9.53 ± 0.09	0.01	0.16	95.67 ± 0.65	0.46	0.61
100 µm	9.48 ± 0.081	0.01	0.11	94.99 ± 0.49	0.26	0.63

**Table 2:** The intergroup comparisons of the linear dimensions and surface area, with the 25 µm, 50 µm, and 100 µm groups at printing time zero (T0), using one-way ANOVA and Tukey’s post hoc.

Layer Thickness of Groups at T0	Surface Area			
	Mean Difference (mm)	Adjusted *p*-Value	Mean Diff. (mm^2^)	Adjusted *p*-Value
25 vs. 50 µm	0.10	0.054	0.10	0.913
25 vs. 100 µm	0.15	0.002	0.78	0.007
50 vs. 100 µm	0.05	0.427	0.68	0.019

**Table 3:** The dimensional stability of linear and surface area over time in the 25 µm, 50 µm, and 100 µm groups, using one-way ANOVA and Tukey’s post hoc. * *p* < 0.05.

Groups Compared at Different Times		Linear Dimension				Surface Area			
		Mean 1st (mm)	Mean 2nd (mm)	Mean Diff. (mm)	Adjusted *p*-Value/ Significance	Mean 1st (mm^2^)	Mean 2nd (mm^2^)	Mean Diff. (mm^2^)	Adjusted *p*-Value/ Significance
*1st*	*2nd*								
**25 T0**	**25 T1**	9.63	9.67	−0.04	0.821	95.76	103.9	−8.09	0.001 *
**25 T0**	**25 T2**	9.63	9.67	−0.04	0.849	95.76	104.6	−8.80	0.001 *
**25 T0**	**25 T3**	9.63	9.72	−0.08	0.230	95.76	103.9	−8.12	0.001 *
**25 T1**	**25 T2**	9.67	9.67	0.00	0.999	103.9	104.6	−0.717	0.791
**25 T1**	**25 T3**	9.67	9.72	−0.05	0.714	103.9	103.9	−0.03	0.999
**25 T2**	**25 T3**	9.67	9.72	−0.05	0.679	104.6	103.9	0.68	0.824
**50 T0**	**50 T1**	9.53	9.66	−0.13	0.001 *	95.67	105.3	−9.61	0.001 *
**50 T0**	**50 T2**	9.53	9.64	−0.10	0.006 *	95.67	105.5	−9.86	0.001 *
**50 T0**	**50 T3**	9.53	9.67	−0.14	0.001 *	95.67	100.5	−4.87	0.001 *
**50 T1**	**50 T2**	9.66	9.64	0.03	0.770	105.3	105.5	−0.25	0.888
**50 T1**	**50 T3**	9.66	9.67	−0.01	0.998	105.3	100.5	4.74	0.001 *
**50 T2**	**50 T3**	9.64	9.67	−0.03	0.670	105.5	100.5	4.99	0.001 *
**100 T0**	**100 T1**	9.48	9.66	−0.18	0.0001 *	94.99	104.5	−9.54	0.001 *
**100 T0**	**100 T2**	9.48	9.61	−0.13	0.001 *	94.99	104.8	−9.86	0.001 *
**100 T0**	**100 T3**	9.48	9.71	−0.22	0.0001 *	94.99	101	−5.99	0.001 *
**100 T1**	**100 T2**	9.66	9.61	0.06	0.229	104.5	104.8	−0.31	0.881
**100 T1**	**100 T3**	9.66	9.71	−0.042	0.478	104.5	101	3.56	0.001 *
**100 T2**	**100 T3**	9.61	9.71	−0.10	0.009 *	104.8	101	3.87	0.001 *

**Table 4:** Surface roughness comparison among the three groups (25, 50, 100 µm) using one-way ANOVA and Tukey’s post hoc.

Group Name and Parameter		Mean 1st	Mean 2nd	Mean Diff.	Adjusted *p*-Value
1st	2nd				
25 *Sa*	50 *Sa*	1.136	0.88	0.25	0.114
25 *Sa*	100 *Sa*	1.136	0.94	0.19	0.265
50 *Sa*	100 *Sa*	0.8838	0.94	−0.06	0.880
25 *Sz*	50 *Sz*	12.82	14.22	−1.4	0.919
25 *Sz*	100 *Sz*	12.82	12.3	0.52	0.988
50 *Sz*	100 *Sz*	14.22	12.3	1.92	0.853
25 *Str*	50 *Str*	0.32	0.46	−0.14	0.478
25 *Str*	100 *Str*	0.32	0.59	−0.27	0.080
50 *Str*	100 *Str*	0.46	0.59	−0.13	0.536
25 *Spc*	50 *Spc*	4637	3876	760.40	0.506
25 *Spc*	100 *Scp*	4637	3709	928.30	0.367
50 *Spc*	100 *Scp*	3876	3709	167.90	0.966
25 *Sdr*	50 *Sdr*	0.48	0.54	−0.05	0.954
25 *Sdr*	100 *Sdr*	0.48	0.35	0.13	0.752
50 *Sdr*	100 *Sdr*	0.54	0.35	0.18	0.575

## Data Availability

The data presented in this study are available on request from the corresponding author.
